# Electric stimulation of the vagus nerve reduced mouse neuroinflammation induced by lipopolysaccharide

**DOI:** 10.1186/s12950-016-0140-5

**Published:** 2016-10-29

**Authors:** G. Meneses, M. Bautista, A. Florentino, G. Díaz, G. Acero, H. Besedovsky, D. Meneses, A. Fleury, A. Del Rey, G. Gevorkian, G. Fragoso, E. Sciutto

**Affiliations:** 1 Instituto de Investigaciones Biomédicas, Universidad Nacional Autónoma de México, AP 70228, Circuito Escolar S/N, Coyoacán, CP 04510 Ciudad de México Mexico; 2 Institute of Physiology and Pathophysiology, Medical Faculty, Philipps University, Marburg, Germany; 3 Unidad Periférica, Instituto de Investigaciones Biomédicas, UNAM / Instituto Nacional de Neurología y Neurocirugía, Colonia la Fama, Delegación Tlalpan, Ciudad de México Mexico; 4 Facultad Mexicana de Medicina, Universidad La Salle, Fuentes 17, Colonia, Tlalpan, Delegación Tlalpan, C.P. 14000 Ciudad de México Mexico

**Keywords:** Neuroinflammation, Microglia, Lipopolysaccharide neuropathologies, Stimulation of vagus nerve, Antiinflammatory

## Abstract

**Background:**

Neuroinflammation (NI) is a key feature in the pathogenesis and progression of infectious and non-infectious neuropathologies, and its amelioration usually improves the patient outcome. Peripheral inflammation may promote NI through microglia and astrocytes activation, an increased expression of inflammatory mediators and vascular permeability that may lead to neurodegeneration. Several anti-inflammatory strategies have been proposed to control peripheral inflammation. Among them, electrical stimulation of the vagus nerve (VNS) recently emerged as an alternative to effectively attenuate peripheral inflammation in a variety of pathological conditions with few side effects.

Considering that NI underlies several neurologic pathologies we explored herein the possibility that electrically VNS can also exert anti-inflammatory effects in the brain.

**Methods:**

NI was experimentally induced by intraperitoneal injection of bacterial lipopolysaccharide (LPS) in C57BL/6 male mice; VNS with constant voltage (5 Hz, 0.75 mA, 2 ms) was applied for 30 s, 48 or 72 h after lipopolysaccharide injection. Twenty four hours later, pro-inflammatory cytokines (IL-1β, IL-6, TNFα) levels were measured by ELISA in brain and spleen extracts and total brain cells were isolated and microglia and macrophage proliferation and activation was assessed by flow cytometry. The level of ionized calcium binding adaptor molecule (Iba-1) and glial fibrillary acidic protein (GFAP) were estimated in whole brain extracts and in histologic slides by Western blot and immunohistochemistry, respectively.

**Results:**

VNS significantly reduced the central levels of pro-inflammatory cytokines and the percentage of microglia (CD11b/CD45^low^) and macrophages (CD11b/CD45^high^), 24 h after the electrical stimulus in LPS stimulated mice. A significantly reduced level of Iba-1 expression was also observed in whole brain extracts and in the hippocampus, suggesting a reduction in activated microglia.

**Conclusions:**

VNS is a feasible therapeutic tool to attenuate the NI reaction. Considering that NI accompanies different neuropathologies VNS is a relevant alternative to modulate NI, of particular interest for chronic neurological diseases.

## Background

It is widely accepted that inflammation is beneficial for the organism by limiting the infectious process and promoting tissue repair and recovery. However, inappropriate inflammation (in time, place, and/or magnitude) is increasingly implicated in a wide range of pathologies [1]. This dual role of inflammation is also observed in the central nervous system (CNS), where neuroinflammation (NI) may take part in disease resolution [2] but also cause severe damage [3]. NI occurs when microglia, astrocytes (brain-resident cells), and perivascular macrophages at the blood-brain barrier (BBB) are activated. These cells produce and release pro- inflammatory mediators that in turn may recruit components of the adaptive immunity [4]. Altogether, these events may result in myelin and neuron damage with ensuing neuronal dysfunction [3].

It has been shown that the CNS senses peripheral inflammation through different pathways, resulting in central inflammation and in a “sickness behavior” profile that may include depression, anxiety, anorexia, and/or lethargy [5]. This communication could proceed through circumventricular organs [6] as well as by the proper lymphatic system, considering the findings recently published [7]. It is also feasible that cytokines can be transported from the periphery to the CNS across the BBB [8], which may become more efficient if some degree of disruption of this barrier occurs [9].

Systemic injection of bacterial lipopolysaccharide (LPS) into mice has been widely used to induce central inflammation experimentally [10, 11]. A single systemic administration of LPS (5 mg/kg, i.p.), resulted in a rapid activation of brain microglia and an increased expression of brain pro-inflammatory factors, such as TNFα and IL-1β, that remained elevated for 10 months, while peripheral TNFα (serum and liver) declined after 9 h and 1 week in serum and liver, respectively [11].

Multiple mechanisms, including the vagus nerve circuit, have evolved to promote a balanced status between local and systemic inflammation, thus preventing immunopathologies [12]. Through efferent and afferent fibers, the vagus nerve regulates numerous central and peripheral key processes [13]. The ascending afferent vagal nerve fibers can inform the CNS of the presence of peripheral inflammation [14]. It has been proposed that vagal efferent fibers release acetylcholine (Ach), which can interact with α7-subunit-containing nicotinic receptors in tissue macrophages and other immune cells to rapidly inhibit the synthesis/release of pro-inflammatory cytokines such as TNFα, IL-1β, IL-6, and IL- 18 [15–18]. However, the direct role of efferent cholinergic vagal nerve fibers in blunting inflammation has been questioned [19–21] since experimental evidence suggests that electric vagus nerve stimulation (VNS) inhibits inflammation indirectly [20] by eliciting a sympathetic response that can mediate anti- inflammatory and other immunoregulatory effects [22–24].

Independently of the mechanisms underlying the anti-inflammatory properties of VNS, its effect has been extensively confirmed under experimental conditions in inflammatory pathologies like arthritis, stroke, cardiovascular diseases, and lupus [25–27]. These findings, together with the use of VNS in humans to treat refractory partial-onset seizures and severe recurrent refractory depression, give rise to great expectations on its use to ameliorate chronic NI and to contribute to the control of the progression of neurodegenerative diseases [28–30].

Herein, the effectiveness of VNS to control the NI experimentally induced in mice by systemic LPS administration was evaluated.

## Methods

### Mice

C57BL/6 J mice were originally purchased from Jackson laboratories, USA, and further bred in a pathogen-free facility at our Institute. Seven to eight week-old male mice were divided into four groups for LPS kinetics experiments and into five groups to test the effect of VNS. Mice were kept in Plexiglas boxes before the experiments. Food and water were allowed *ad libitum*. After surgery, all mice were housed individually at 22 ± 3 °C with a 12 h light-dark cycle. Experiments were repeated two to four times each.

### Surgery and Electric Vagus Nerve Stimulation (VNS)

**Fig. 1:**
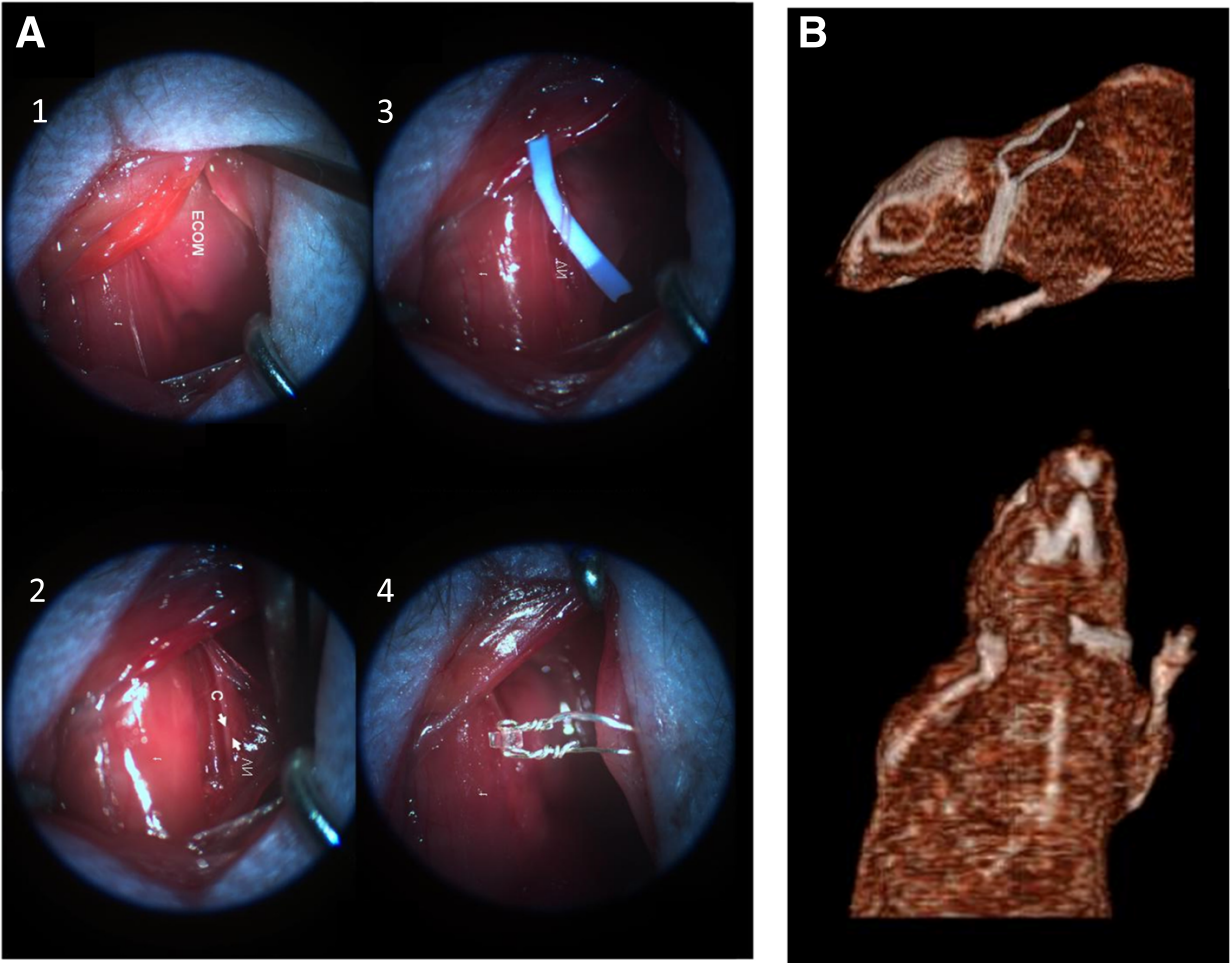
Procedure of implanted electrodes and location by CTscan. **a** Procedure of electrode insertion into the vagus nerve. 1. Photograph showing the trachea and the sternocleidomastoid muscle, carotid. 2. Sheath with carotid and vagus nerve. 3. Vagus nerve is shown isolated. 4. Electrodes applied to the vagus nerve. t = trachea, ECOM = sternocleidomastoid, c = carotid, VN = vagus nerve. **b** Representative view of the electrodes inserted in the vagus nerve by Computed Tomography Scan

To perform its implantation, an incision was performed in the ventral side of the neck of mice to isolate the left cervical vagus nerve, and a platinum electrode was placed under the exposed nerve (Fig. 1a). The view of the implanted electrodes by CTscan is illustrated in Fig. 1b.

All mice were anesthetized with ketamine (90–100 mg/kg) and xylazine (10 mg/per kg). Bipolar electrodes were placed on the cervical vagus nerve trunk and connected to a stimulation module (designed by the Centro de Ciencias Aplicadas y Desarrollo Tecnológico, CCADET, UNAM, Mexico). After electrodes implantation, mice were randomly divided in five different groups that received: group 1, (sham, non-LPS treated and with no electric stimulus); group 2, only treated with isotonic saline solution (ISS); group 3, only VNS treated; group 4, only treated with LPS and group 5, LPS and VNS treated. The parameters for VNS were calculated based on previously reported data [31]. VNS stimulated anesthetized mice received a constant voltage of 5 Hz, 0.75 mA, for 30 s.

### LPS-induced inflammation

**Fig. 2:**
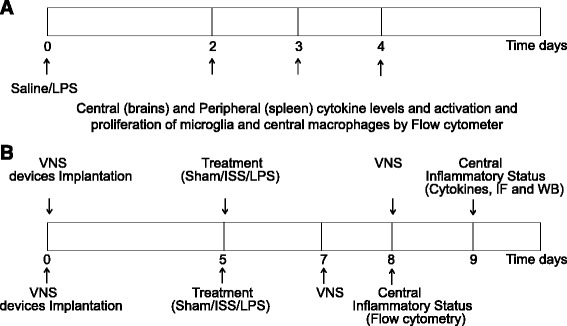
Experimental design line. **a** LPS or ISS were injected at day 0. The inflammatory peripheral and central status was evaluated before and at the different times after injection. **b** Electrodes were implanted at day 0. Five days later, mice were randomly divided into five groups: sham, isotonic saline solution treated mice (ISS), Vagus Nerve Stimulation (VNS), LPS and LPS + VNS

A model of CNS inflammation induced by systemic LPS administration was used, as previously described [11]. Mice received either 5 mg/kg of LPS from *Escherichia coli* serotype 0111:B4 (Sigma, St. Louis, MO) injected intraperitoneally (i.p.) or an equivalent volume of saline solution vehicle (ISS), 0.9 % NaCl (endotoxin-free isotonic saline solution) (PiSA, Mexico) as a control. To determine the best point time that LPS induces inflammation, the peripheral and central inflammatory response was assessed before and 2, 3, and 4 days after LPS administration (Fig. 2a).

### Central and Peripheral Inflammatory mediators

Mice were anesthetized as described above, and submandibular bleeding was performed before and at different times after LPS treatment (Fig. 2a). LPS- treated mice were employed to evaluate the VNS as an anti-neuroinflammatory treatment (Fig. 2b). All mice were perfused by cardiac puncture with 250 mL of sterile NaCl 0.15 M to prevent the presence of peripheral molecules in central tissues. Brains were rapidly removed and half of them were processed for protein extraction to quantify the levels of TNFα, IL-1β, and IL-6 and for western blot analysis. The other half of the brains were fixed for immunofluorescence analysis as described below. Only for the LPS kinetic experiment (Fig. 2a), spleens were removed to estimate the level of peripheral inflammatory soluble mediator because they offer more material than the insufficient amount of sera of each mouse to measure these mediators.

### Protein extraction

Snap-frozen brain hemispheres and spleens were homogenized in a lysis buffer (50 mM HEPES, 150 mM NaCl, 1 % Nonidet-p40, 0.5 % sodium deoxycholate, 0.1 % SDS) containing complete protease inhibitors (Roche, IN). Samples were then centrifuged at 16,000 × *g* for 15 min at 4 °C and supernatants were collected for analysis. The total amount of proteins in the soluble extract was measured using the Lowry method [32].

### Cytokine Enzyme-Linked Immunosorbent Assay (ELISA)

Commercial kits were used to quantify the concentration of the pro-inflammatory cytokines IL-1β, IL-6, and TNFα in brain and spleen extracts (BioLegend, San Diego, CA). Briefly, sandwich ELISAs were performed in 96-well, flat-bottom MaxiSorp microtiter plates (Nunc, Roskilde, Denmark). Microplates were coated with the capture antibody for 18 h at 4 °C. After washing with PBS-Tween-20 (0.05 %) and blocking for 60 min at room temperature with 2 % PBS-BSA, plates were incubated at room temperature for 2 h with standard or samples, washed three times, and incubated for 1 h with the detection antibody at room temperature.

Bound detection antibodies were identified using 1:1000 diluted Avidin-HRP and TMB as a substrate. Optical density was read before and after the reaction was stopped with H_2_SO_4_ 2 N at 450 and 630 nm, respectively. Results were expressed in pg/mL per mg of protein in the respective soluble extract.

### Western blot

Proteins were separated by electrophoresis on 12 % Tris-glycine polyacrylamide gel at 150 V for 1 h 30 min, (for Iba-1 detection) and on 4–12 % polyacrylamide precast NuPAGE Bis-Tris gel (Invitrogen) at 200 V for 2 h 30 min (for GFAP detection), and transferred onto a PVDF membrane (Bio-Rad, Hercules, CA) using a semi-dry blot system (Bio-Rad) at 25 V for 50 min. Membranes were blocked in PBS/2 % fat-free dry milk overnight at 4 °C and then incubated with primary antibodies (rabbit anti-β- actin and rabbit anti-GFAP [1:1000] or rabbit anti-Iba-1 [1:2000]) diluted in PBS/milk/0.2 % Triton X-100 overnight at 4 °C. After washing with PBS/0.2 % Tween, membranes were incubated with HRP-conjugated anti-rabbit IgG (Invitrogen) for 2 h at room temperature. Immunoreactive bands were detected by chemiluminescence using the Super Signal West Dura Extended Duration Substrate kit (Pierce, IL). The NIH ImageJ software was used for densitometric analysis of bands. β-actin served as an internal control.

### Immunofluorescence Analysis (IFC)

The half of each brain obtained as described above was fixed in 4 % buffered formalin at 4 °C overnight. Free-floating 30 μm-thick mouse brain sections were processed as previously described [33]. After antigen retrieval by incubating in citrate buffer (0.01 M citric acid, 0.05 % Tween 20, pH 6.0) at 70 °C for 50 min, samples were thoroughly washed several times with TBS and blocked with a solution of 2 % IgG-free albumin (Sigma) in TBS for 20 min at room temperature. Brain sections were then incubated overnight at 4 °C with rabbit anti-GFAP (Invitrogen) or anti-Iba-1 (WAKO, VA) polyclonal antibody in TBS-2 % BSA to detect astrocytes or microglia, respectively. After washing, sections were incubated for 1 h at room temperature with AlexaFluor 594 goat anti-rabbit IgG (Molecular Probes, OR) diluted in TBS-2 % BSA. Samples were mounted onto glass slides in Vectashield medium (Vector Laboratories, CA) containing DAPI for nuclei imaging. Samples were viewed on an Olympus Ix51 microscope equipped with a DP71 camera (Nikon Instruments Inc., NY).

### Brain cells Isolation

Parallel experiments in other groups of mice were performed for Flow cytometry analysis. Mice were anesthetized with a mixture of ketamine and xylazine, as described above. Brain cells isolation was obtained following a method previously reported, with minor modifications [34, 35]. Briefly, mice were perfused by cardiac puncture with 250 mL of sterile GKN approximately (8 g/L NaCl, 0.4 g/L KCl, 3.56 g/L Na_2_HPO_4_∙12H_2_O, 0.78 g/L NaH_2_PO_4_∙2H_2_O, 2 g/L D-(+)-glucose, pH 7.4) to remove all peripheral cells of the central tissues. Brains were recovered in ice-cold GKN containing 0.02 % (w/v) isotonic bovine seroalbumin (GKN-BSA), and mechanically dissociated using a 100-μm mesh. The sediment was collected in a 50-mL centrifuge tube, washed with GKN-BSA, and centrifuged at 400 × *g* for 10 min at slow brake. The pellet was treated with 5 mL digestion buffer (4 g/L MgCl_2_, 2.55 g/L CaCl_2_, 3.73 g/L KCI, and 8.95 g/L NaCl, pH 6.7) supplemented with 15 U of type-II collagenase and 500 U of DNase I per brain for 1 h at 37 °C, washed with GKN-BSA, and centrifuged at 400 × *g* for 10 min at slow brake.

The pellet obtained was resuspended in 4 mL 30 % Percoll with GKN-BSA and placed on a gradient of 70 % and 37 % of Percoll, centrifuged at 500 × *g* for 20 min and resuspended in PBS (1X-0.02 % NaN_3_). Cells were counted, stained with specific antibodies and analyzed by flow cytometry.

### Flow cytometry

Isolated brain cells were treated with CD16/32 antibody to block Fc receptors and stained for surface markers as previously reported [35]. Cells were labeled with anti CD11b-FITC and anti-CD45-APC or isotype-matched control antibodies (BioLegend) and analyzed using a FACSCalibur flow cytometer and the Cell-Quest Pro software. For analysis, two cell populations were distinguished using antibodies against CD11b and CD45. As both microglia cells and macrophages are CD11b positive, the difference in the level of CD45 expression was used to distinguish between microglia, which expresses low CD45 levels, and macrophages, that express high CD45 levels [36]. Microglia and macrophage activation status was assessed by examining the increase in medium fluorescence intensity for CD11b + in CD11b+/CD45^low^ and CD11b+/CD45^high^ cell population [37].

### Statistical analysis

All analyses were carried out using Instat Statistical software (GraphPad Software Inc., La Jolla, CA, USA). Data are reported as mean ± standard error (SEM).

Differences between groups were evaluated using the Kruskal-Wallis test (non- parametric ANOVA test) or one-way Analysis of Variance plus the Tukey-Kramer multiple comparisons test, considering the distribution of the data, as described in each Table and Figure.

## Results

### Effect of LPS on Peripheral and Central Pro-Inflammatory Cytokine Levels

To identify the number of days after LPS treatment in which levels of NI parameters were adequate to evaluate the anti-NI effects of VNS, peripheral and central levels of pro-inflammatory cytokines (IL-6, IL-1β, and TNFα) were analyzed after LPS administration. As show in Table 1, a statistically significant increase in central TNFα levels was observed 4 days after LPS injection. IL-6 was found elevated 1 and 4 days after LPS injection. IL-1β was increased from the second day after LPS injection and remained elevated until day four. As expected, no significant differences in the levels of peripheral cytokines were found after LPS treatment at these time points (data not shown).

**Table 1:** Cytokine levels (pg/mg of protein) in brain extracts from ISS- or LPS-treated mice

	TNFα	IL-1β	IL-6
ISS	0.24 ± 0.04^a^	4.9 ± 1.1^a^	0.2 ± 0.6^a^
Days after LPS treatment:			
2	0^b^	31.5 ± 4.3^a^	1.9 ± 1.4^a^
3	0^b^	18.8 ± 3.3^a,b^	0.2 ± 0.2^a^
† 4	3.5 ± 0.4^a,c^	112 ± 19.2^c^	46.2 ± 4.6^b^

Mean ± SEM of brain cytokine levels in saline- or LPS-treated C57BL/6 J male mice (*n* = 5, † one mouse dead after LPS injection). Different literals ^a,b,c^indicate significant differences in the level of each cytokine between saline-treated and 2, 3, and 4 days post-LPS treated mice using the Kruskal-Wallis test (non-parametric ANOVA), TNFα (*p* = 0.0004), IL-1β (*p* = 0.0003), IL-6 (*p* = 0.008). Data are from one out of three experiments

### Effect of LPS on Brain Microglia and Macrophages

**Fig. 3:**
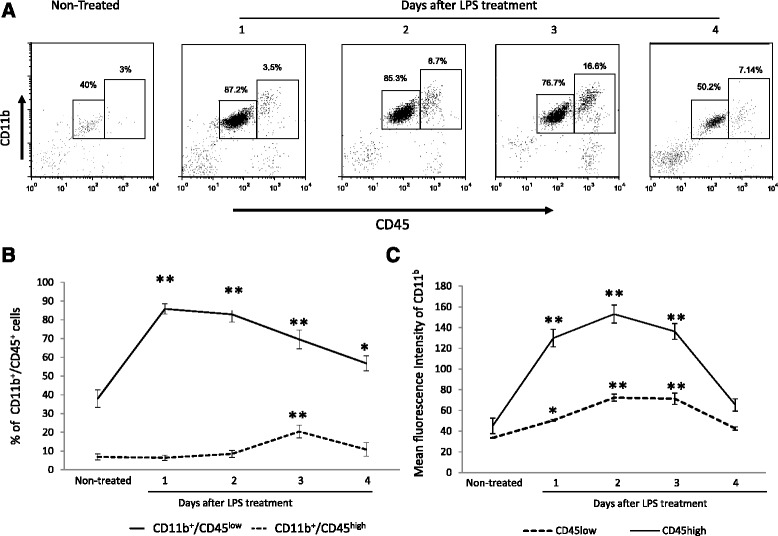
**a** A representative dot plot of isolated brain cells from saline or LPS treated mice analyzed by flow cytometry. **b** Mean ± SD of the percentage of CD11b/CD45^low^ and CD11b/CD45^high^ before and 48, 72, and 96 h after LPS treatment and mean ± SD of the fluorescence intensities of CD11b (**c**). Different literals indicate significant differences between the different groups (*p* < 0.05) using the Kruskal-Wallis test (non-parametric ANOVA) plus the Dunn’s multiple comparisons test. Data are representative of four experiments

To evaluate the central cellular inflammatory effects of systemic LPS injection, the percentage of CD11b^+^/CD45^low^ (microglia) and CD11b^+^/CD45^high^ (macrophages) were estimated daily for 3 days after LPS injection starting at day 2. As shown in Fig. 3, the relative percentage of microglial cells and macrophages was increased from day 2 to day 3 after LPS treatment lowering by day 4. Activation of the microglia e.g. increased in the mean intensity fluorescence of CD11b was found at day two and three whilst activation of macrophages was observed only at day two after LPS injection.

### Effect of VNS on the Central Expression of Cytokines

After electrodes were placed on the cervical vagus as described in Materials and Methods (Fig. 1), mice were randomized into five groups: sham and four experimental groups, treated with ISS or LPS or VNS or LPS + VNS (electrode stimulation was applied only for those described as VNS).

Since the concentration of IL-6, IL-1β, and TNFα showed significant changes on day four after LPS injection only at central levels, these cytokines were measured in the brain extracts 1 day after VNS (Fig. 2b). As shown in Table 2, increased central levels of the three cytokines were detected in LPS-treated mice. Twenty- four hours after VNS, a statistically significant decrease in the level of the three cytokines was observed compared to the LPS-treated group. No effect on the level of the three cytokines was observed in sham or VNS-treated mice.

**Table 2:** Cytokine (pg/mg of protein) levels in brain extracts of sham and treated mice

	TNFα	IL-1β	IL-6
Sham	0.94 ± 0.25^a^	10.54 ± 3.7^a^	0.78 ± 0.20^a^
ISS	0.34 ± 0.3^a^	4.1 ± 0.7^a^	0.62 ± 0.03^a^
VNS	0.06 ± 0.06^a^	7.4 ± 3.8^a^	0.28 ± 0.06^a^
††LPS	3.7 ± 0.38^b^	184 ± 1.87^b^	22.3 ± 0.72^b^
†LPS + VNS	1.47 ± 0.04^a^	20.4 ± 1.55^a^	1.19 ± 0.08^a^

Mean ± SEM of brain cytokine levels in groups of five C57BL/6 male mice each († one and †† two mice dead after LPS injection). VNS and LPS + VNS reported levels of central cytokines 24 h after VNS treatment in mice injected or not with LPS 3 days before (Fig. 2b). Different literals indicate significant differences in each cytokine level between the different treatments using the Kruskal-Wallis test (non- parametric ANOVA), TNFα (*P* = 0.025), IL-1β (*P* = 0.004), IL-6 (*P* = 0.005). Data are from one out of four experiments

### Effect of VNS on CD11b/CD45^low^ and CD11b/CD45^high^ Brain Cells

Considering the results obtained in the kinetic study (Fig. 3), 2 days after ISS or LPS treatment, the vagus of the mice were electrically stimulated and the effect on microglial cells and macrophages was evaluated 24 h later (Fig. 2b). LPS significantly increased the percentage of CD11b+/CD45^low^ and CD11b+/CD45^high^cells.

Electric stimulation of the vagus nerve did not significantly affect the percentage of CD11b+/CD45low cells as compared to that of non-treated or saline-injected animals.

**Fig. 4:**
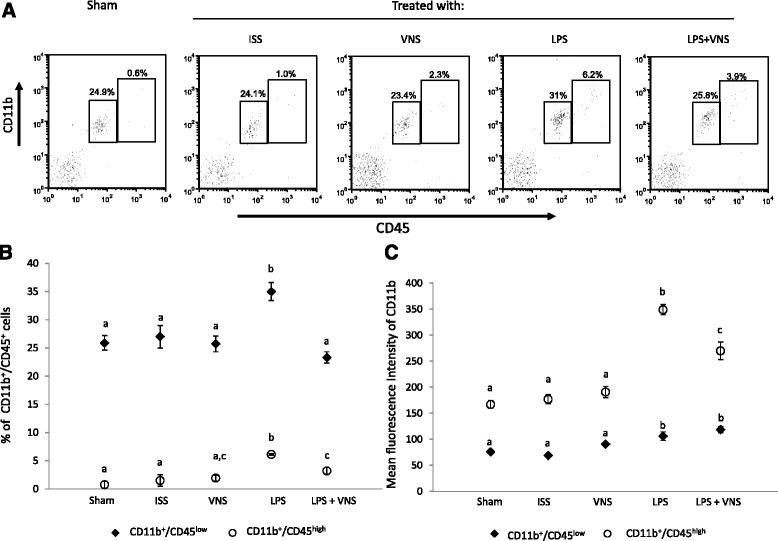
**a** Representative dot plot of isolated brain cells analyzed by flow citometry. **b** Mean ± SD of the percentage of CD11b/CD45^low^ and CD11b/CD45^high^ cells and the respective Mean ± SD of the fluorescence intensities of CD11b (**c**) of five mice per group. Data are representative of two different independent assays. Different literals indicate significant differences in the percent of CD11b/CD45^low^ and CD11b/CD45^high^ cells (*P* < 0.05) between the different groups using the Kruskal- Wallis test (non-parametric ANOVA) plus the Dunn’s multiple comparisons test

The percentage of CD11b+/CD45^high^ cells also decreased after VNS treatment but in a lower extent (Fig. 4b). The mean intensity of fluorescence of CD11b in CD11b+/CD45^low^ and CD11b+/CD45^high^ windows, was also found significantly increased in LPS-treated mice and was reduced in a statistically significant manner by VNS (Fig. 4c).

### Effect of VNS on Iba-1 and GFAP Expression

**Fig. 5:**
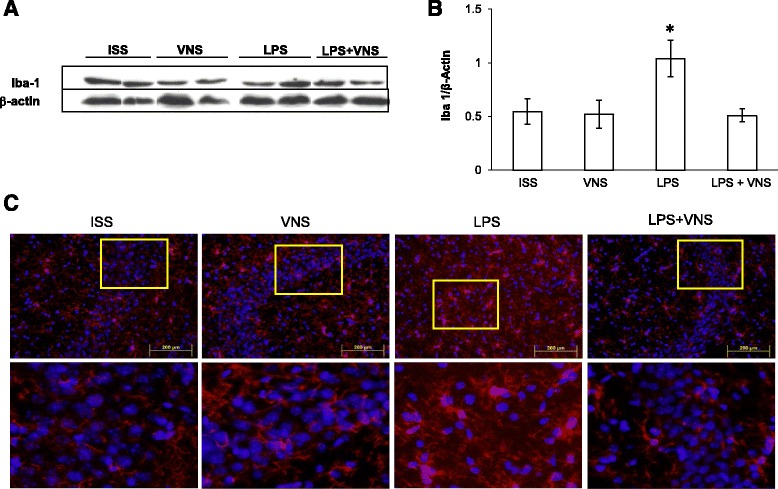
Analysis of brain Iba-1 expression in ISS, VNS, LPS, and LPS + VNS- treated mice. **a** Representative Western blot showing Iba-1 and β-actin. **b** Western blot analysis of Iba-1 in whole brain homogenates of SSI, VNS, LPS, and LPS + VNS-treated mice. Each column represents the level of Iba-1 expressed as mean ± SEM, normalized to β-actin in a same gel. Different literals indicate significant differences in the expression level of Iba-1 among the different groups using one-way Analysis of Variance plus the Tukey-Kramer multiple comparisons test. *F* (3,16) = 6.7, *p* < 0.004. **c** Representative mouse brain sections of the different groups stained with anti-Iba-1 antibody (to detect microglia). Bottom images represent a nine-fold magnification of the region outlined in the box in the corresponding upper image. In LPS-treated mice, higher numbers of microglia with morphological characteristics of activated cells were observed

To further characterize the effect of peripheral LPS administration and VNS on microglial activation, a Western blot assay using brain homogenates was performed. Increased levels of Iba-1, a microglia marker, were found in LPS- treated mice with respect to ISS-treated. Importantly, VNS significantly reduced the expression of Iba-1 (Fig. 5a and b). To characterize eventual morphological changes in microglia, the expression of Iba-1 was evaluated by immunohistochemistry. Four days after LPS treatment, microglial cells with retracted processes and enlarged cell bodies, some with curved contour, were observed after staining with anti-Iba-1 antibody (Fig. 5c). Iba-1 expression was significantly increased in animals of the LPS compared to mice from ISS, VNS and LPS + VNS groups (Fig. 5c).

**Fig. 6:**
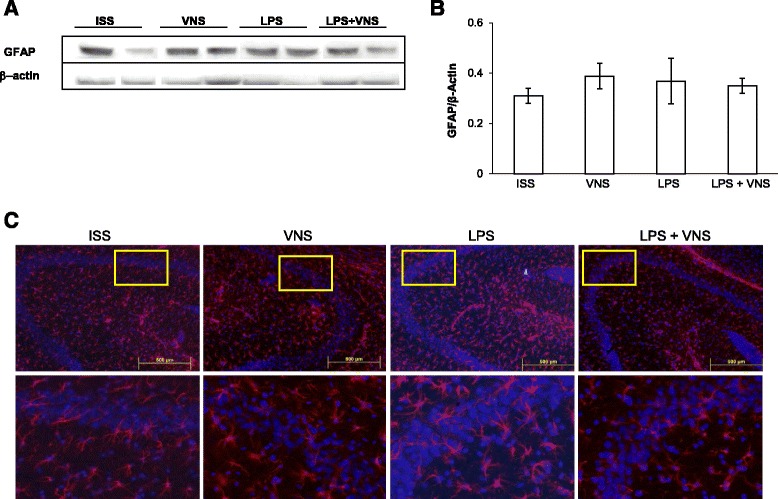
Analysis of GFAP expression in the brain of ISS, VNS, LPS-treated and LPS + VNS-treated. **a** Representative Western blots showing GFAP and β-actin levels. **b** Western blot analysis of GFAP in whole brain homogenates of untreated, VNS, LPS-treated, and LPS + VNS-treated mice. Each column in the graph represents the level of GFAP expressed as Mean ± SEM, normalized to β-actin in a same gel. No significant differences between GFAP levels were observed using one-way Analysis of Variance plus the Tukey-Kramer multiple comparisons test. *F* (3,16) = 0.44, *p* = 0.72. **c** Representative mouse brain sections of the different groups of mice stained with anti-GFAP antibody (to detect astrocytes). Bottom images represent a nine-fold magnification of the region outlined in the box in the corresponding upper image

As shown in Fig. 6, no significant differences in GFAP expression in astrocytes were observed among experimental groups (as determined by Western blot and immunohistochemistry using an anti-GFAP antibody). These results are in agreement with previous reports [38].

## Discussion

VNS has been found efficacious in controlling LPS-induced acute peripheral inflammation [15]. VNS also attenuates the chronic systemic inflammation that accompanied different experimental inflammatory diseases such as mouse arthritis [39], rat heatstroke [40], and rat pulmonary inflammation [41] through vagal anti- inflammatory pathway. There is also evidence that VNS also limits the central influx of activated T cells in the lesioned facial motor nucleus [42] and also reduces the central inflammatory response in the murine experimental autoimmune encephalomyelitis [43]. It was recently reported that VNS also control the disease severity and inflammation in severe human rheumatoid arthritis [44].

In this study, clear evidence of the anti-neuroinflammatory effect of the VNS is shown. While the anti-inflammatory effect of VNS exerted via α7-nicotine cholinergic receptor stimulation, is well established our studies showed for the first time that this procedure inhibits IN.

The increased central levels of pro-inflammatory cytokines induced by peripheral LPS injection were significantly reduced after VNS treatment. The percent of microglia (CD11b+/CD45^low^) was also significantly reduced after VNS in LPS-treated mice (Fig. 4b). In agreement with these findings, a reduction in the levels of Iba-1, a calcium-binding protein specifically expressed in microglia/macrophages, was also observed [38]. Considering that microglia proliferation is part of the activation process, it is feasible that activation occurred earlier after LPS treatment [45, 46]. It is also interesting to note that VNS significantly decreases the percentage and the activation of central macrophages CD11b+/CD45^high^ (Fig. 4).

As shown in Fig. 5, a decrease in Iba-1 expression was demonstrated by Western blot and by immunohistochemistry in the hippocampus of VNS-treated mice, a finding that supports the effective control of microgliosis by this treatment. This effect on key resident immune cells of the CNS is critical in controlling the neuroinflammatory response. Microglia activation has a significant effect on NI progression, since glial cells are the primary source of pro-inflammatory cytokines in the brain. In addition, recent evidence point to the significant contribution of microglial proliferation in the progression of a chronic neurodegeneration induced in a murine model of prion disease [47]. Interestingly in the latter study low levels of inflammatory cytokines were detected, suggesting that microglial proliferation is implicated in the progression of the disease without direct participation of inflammatory cytokines. On the other hand, in our study astrocytes were not activated 4 days after LPS administration, irrespective of whether mice were given VNS or not. Our results are in agreement with previous reports where authors did not observe differences in GFAP immunoreactivity at any time point after one or four repeated peripheral LPS challenge [38]. The western blot assays for evaluation of Iba-1 and GFAP levels (Figs. 5 and 6) were performed using whole brain homogenates. Considering that the GFAP glial protein is heterogeneously expressed in astrocytes from different brain regions and is extremely low in the cortex [48], its expression in the hippocampus was analyzed and no differences were observed between experimental groups (data not-show).

Another point that merits comments is the low frequency stimulation protocol used in this study for VNS that favors the activation of the efferent rather than afferent fibers of the VN [49].

Altogether, the findings reported here, let us to propose VNS as a novel and useful alternative to modulate NI. This could be especially relevant since treating NI remains an unsolved challenge. NI is a hallmark of several infectious and neurodegenerative diseases [50–53], and its control could result in a clear improvement of the patients [54]. However, the available anti-inflammatory tools are not appropriate for chronic treatments. Glucocorticoids (GCs), particularly synthetic GCs like dexamethasone and prednisone, are the drugs of choice to treat NI. While both are highly effective immunosuppressive drugs to reduce NI, long-term treatment is limited by side effects due to the high systemic doses required to reach effective concentrations in the CNS [55]. In contrast, VNS could be a much safer alternative for chronic NI control. VNS has been applied for drug-resistant epilepsy for 17 years with a good safety record [56]. Moreover, it was approved for this indication by the US Food and Drug Administration in 1997 and in 2001 in Europe and Canada. Since then, its use in neuromodulation for epilepsy control has spread. Over 100,000 patients have been implanted until December 2012 with a VNS device (“Cyberonics announces 100,000th patient implant of VNS therapy”, ir.cyberonics.com/releasedetail.cfm?ReleaseID = 728198) [57]. Its usefulness has also been explored to treat neurodegenerative diseases with some success [58].

Finally, it is worthy to mention that the wide acceptance of this new procedure to control chronic NI will require further investigation to support its usefulness in different pathological conditions. Considering the minor undesirable side effects of this intervention, some work aiming at reducing the cost of the electronic device would be relevant, since it would offer a good therapeutic choice in non-developed countries.

## Conclusions

In conclusion, this study provides clear evidence that electric stimulation of the vagus nerve attenuates the inflammatory response in the CNS induced by peripheral LPS challenge. To our knowledge, this is the first study in which the usefulness of VNS to control the NI is reported. Albeit the mechanism that underlie the anti-neuroinflammatory effects induced by VNS remains to be elucidated, results shown herein open a new therapeutic alternative of special interest to reduce the NI involved in the evolution of several chronic neuropathologies.

## Abbreviations

Ach: Acetylcholine
BBB: Blood-brain barrier
BSA: Isotonic bovine seroalbumin
CNS: Central nervous system
GFAP: Glial fibrillary acidic protein
GKN: Glucose–potassium–sodium
Iba-1: Ionized calcium binding adaptor molecule 1
ip: Intraperitoneally
LPS: Lipopolysaccharide
NI: Neuroinflammation
TMB: 3,3’5,5’ Tetrametil-bencidina
VNS: Electrical stimulation of the vagus nerve
